# Computational Design and Biological Evaluation of Analogs of Lupin Peptide P5 Endowed with Dual PCSK9/HMG-CoAR Inhibiting Activity

**DOI:** 10.3390/pharmaceutics14030665

**Published:** 2022-03-18

**Authors:** Carmen Lammi, Enrico M. A. Fassi, Jianqiang Li, Martina Bartolomei, Giulia Benigno, Gabriella Roda, Anna Arnoldi, Giovanni Grazioso

**Affiliations:** Dipartimento di Scienze Farmaceutiche, Università degli Studi di Milano, Via L. Mangiagalli 25, 20133 Milan, Italy; enrico.fassi@unimi.it (E.M.A.F.); jianqiang.li@unimi.it (J.L.); martina.bartolomei@unimi.it (M.B.); giulia.benigno@studenti.unimi.it (G.B.); gabriella.roda@unimi.it (G.R.); anna.arnoldi@unimi.it (A.A.)

**Keywords:** PCSK9, HMG-CoA reductase, dual activity, hypercholesterolemia, peptide, lupin, drug design, MM-GBSA

## Abstract

(1) Background: Proprotein convertase subtilisin/kexin 9 (PCSK9) is responsible for the degradation of the hepatic low-density lipoprotein receptor (LDLR), which regulates the circulating cholesterol level. In this field, we discovered natural peptides derived from lupin that showed PCSK9 inhibitory activity. Among these, the most active peptide, known as P5 (LILPHKSDAD), reduced the protein-protein interaction between PCSK9 and LDLR with an IC_50_ equals to 1.6 µM and showed a dual hypocholesterolemic activity, since it shows complementary inhibition of the 3-hydroxy-3-methylglutaryl coenzyme A reductase (HMG-CoAR). (2) Methods: In this study, by a computational approach, the P5 primary structure was optimized to obtain new analogs with improved affinity to PCSK9. Then, biological assays were carried out for fully characterizing the dual cholesterol-lowering activity of the P5 analogs by using both biochemical and cellular techniques. (3) Results: A new peptide, P5-Best (LYLPKHSDRD) displayed improved PCSK9 (IC_50_ 0.7 µM) and HMG-CoAR (IC_50_ 88.9 µM) inhibitory activities. Moreover, in vitro biological assays on cells demonstrated that, not only P5-Best, but all tested peptides maintained the dual PCSK9/HMG-CoAR inhibitory activity and remarkably P5-Best exerted the strongest hypocholesterolemic effect. In fact, in the presence of this peptide, the ability of HepG2 cells to absorb extracellular LDL was improved by up to 254%. (4) Conclusions: the atomistic details of the P5-Best/PCSK9 and P5-Best/HMG-CoAR interactions represent a reliable starting point for the design of new promising molecular entities endowed with hypocholesterolemic activity.

## 1. Introduction

Hyperlipidemia is a well-known risk factor for developing cardiovascular disease [1]. The most common drugs for hypercholesterolemia treatment are statins, which inhibit 3-hydroxy-3-methylglutaryl coenzyme A reductase (HMG-CoAR), the rate-limiting enzyme in cholesterol biosynthesis. This enzyme lowers intracellular cholesterol levels, leading to an increased expression of LDL receptors (LDLR) on cell surfaces and a reduction of serum LDL-cholesterol (LDL-C) via the activation of the sterol-regulatory element-binding protein (SREBP)-2 transcription factor pathway [2]. Although this approach is considered an efficient way to reduce circulating LDL-C, cardiovascular events still occur in some patients. Moreover, statins induce known and serious side effects, such as headache, muscle and joint pain, and a higher risk of developing diabetes.

Another key player in the cholesterol homeostasis is the proprotein convertase subtilisin/kexin 9 (PCSK9) which was discovered in the 2003 and it has been recognized as one of the most promising targets for counteracting hypercholesterolemia and atherosclerotic cardiovascular diseases [3]. Under physiological conditions, the blood-circulating PCSK9 interacts with the low-density lipoprotein receptor (LDLR) on the liver cell membrane, triggering the internalization of the LDLR/PCSK9 complex in a digestive vacuole [4]. Consequently, the main biological activity of PCSK9 is the regulation of the LDLR population on the liver cell surface, resulting in the tuning of the cellular capacity to capture circulating LDL cholesterol (LDL-C). Accordingly, the inhibition of the PCSK9/LDLR interaction leads to an increased LDLR population on the cell membrane, resulting in an enhanced capacity to capture the blood-circulating LDL-C by liver cells [5]. 

The expression of PCSK9 is also controlled by the activity of SREBP-2 as well as a specific transcriptional activator hepatocyte nuclear factor-1α (HNF-1α) [6], which is a liver-enriched transcription factor regulating many target [7] genes in the liver and intestine. In contrast, the ability of SREBP-2 to co-stimulate the PCSK9 and LDLR expression limits the therapeutic efficacy of statins which are known to produce their effects via SREBP-2 activation. Indeed, it is well documented that statins improve the PCSK9 protein level production through the augmentation of the intracellular HNF-1α levels [8].

Hence, in the last two decades, academia and pharmaceutical companies have financed considerable research on the development of compounds capable of target PCSK9 developing different strategies including siRNA, anti-sense oligonucleotides (ASOs), peptide inhibitors, and monoclonal antibodies (mAbs) [5,9,10,11,12,13,14,15,16,17,18]. In this field, we have recently sorted out the most potent natural peptide (LILPKHSDAD, P5) derived from a peptic lupin (*Lupinus A.*) protein hydrolysate [19] with hypocholesterolemic activity [20], which impairs the PCSK9-LDLR interaction with an IC_50_ value of 1.6 µM [21]. In parallel, P5 reduces the catalytic activity of HMG-CoAR with an IC_50_ value of 147.2 µM [22]; through the inhibition of the enzyme activity, P5 increases the LDLR protein level in HepG2 cells through the activation of SREBP-2, and through a downregulation of HNF-1α, it reduces the PCSK9 protein levels and its secretion in the extracellular environment [22]. This unique synergistic multi-target inhibitory behavior of P5 determines the improved ability of HepG2 cells to uptake extracellular LDL with a final hypocholesterolemic effect. P5 was successfully transported by differentiated human intestinal Caco-2 cells [23] through transcytosis [24], and, during transport, it was partially metabolized in a breakdown fragment (LPKHSDAD, P5-met), which retained the biological activity of the parent peptide [24]. In facts, we have demonstrated that P5-met reduces PCSK9-LDLR binding with a dose-response trend and an IC_50_ of 1.7 μM and inhibits the HMG-CoAR with an IC_50_ of 175.3 µM [24]. At a cellular level, such as P5, P5-met improves the LDLR and reduces PCSK9 levels, through the modulation of both SREBP-2 and HNF-1α, respectively [24]. Therefore, since P5-met displayed the same activity and behavior of the parent peptide, P5, our results indicated that the first two amino acid residues (LI) do not play a key role in the interaction with both PCSK9 and HMG-CoAR target.

These evidences clearly indicate that P5, with its dual-inhibitory activity, represents a new alternative strategy to the use of single classical PCSK9 and HMG-CoAR inhibitors. Notably, the strategy in which dual-inhibitors are employed may be more effective in overcoming the deficits attributed to the classical use of statins (adverse effects and co-stimulation of PCSK-9 and LDLR via a common transcriptional activator, i.e., SREBP-2, in statin-treated patients limited the efficacy of these classical HMG-CoAR inhibitors) or PCSK9 inhibitors (including expensiveness, low compliance of the patients, repeated administrations, and injection site irritations) on health and to meet the desired health goals and public priorities in terms of safety and cost-related issues. 

Considering these observations, the overall aim of the present study is the development of new P5 analogs able to target both PCSK9 and HMG-CoAR, therefore displaying an improved and dual hypocholesterolemic activity. To achieve this objective, new P5 analogs with improved PCSK9 and HMG-CoAR inhibitory activity were computationally designed [25]. Hence, the theoretical study was validated and confirmed by performing a detailed biological investigation on the most promising P5 analogs. Firstly, their ability to inhibit the protein–protein interaction (PPI) between PCSK9 and LDLR and the HMG-CoAR activity were evaluated using biochemical assays, respectively. Then, their effects on the cholesterol pathway modulation on HepG2 cells were deep characterized, fostering their dual inhibitory cholesterol-lowering activity. More in details, the effect of PCSK9 inhibition by P5 analogs on the LDLR protein levels on the surface of hepatocytes and their effect to improve the functional ability of hepatocytes to uptake LDL from the extracellular environment were assessed by performing in cell-western (ICW) [26] and LDL-uptake assay [27], respectively, in the presence of PCSK9. In parallel, to assess the effects of P5 analogs on the cholesterol pathway upon HMG-CoAR inhibition, western blotting was performed, monitoring the LDLR, SREBP-2, HMG-CoAR, PCSK9, and HNF-1α protein levels, respectively. In addition, by performing ELISA experiments, the effects of P5 analogs on the hepatic secretion of PCSK9 levels were assessed. Finally, LDL-uptake and ICW assays were carried out to investigate the functional ability of hepatic cells to absorb extracellular LDL upon treatment with P5 derived peptides.

## 2. Materials and Methods

### 2.1. System Setup and MD Simulations

The computational models utilized in this study were built starting from the coordinates of the PCSK9/P5 complex model previously reported by us [21]. Here, the starting PCSK9/P5 complex model was additionally equilibrated through 1 µs-long MD simulations, utilizing the pmemd.cuda module of the AMBER20 package [28]. In particular, the ff14SB AMBER force field [29] was used for simulating the protein atoms, while the TIP3P model [30] was used to explicitly represent the water molecules (about 25,000). The sodium ions were added to neutralize the overall charge of the simulation system and the MD trajectories acquired during the production runs were examined by visual inspection by means of VMD [31], ensuring that the thermalization did not cause any structural distortion. This protocol was also utilized to perform MD simulations on the PCSK9 complexes resulting from the mutations of the P5 sequence.

### 2.2. Cluster Analysis

The MD trajectory frames were analyzed by clustering the conformations adopted by the small peptide backbone atoms in complex with PCSK9. The cluster analysis was performed using the cpptraj module [32] of AMBER20 [28]. By this algorithm, the MD frames were divided into clusters by the complete average linkage algorithm, and the PCSK9/peptide complex conformations with the lowest root mean square deviation (RMSD) to the cluster centers (the structures representative of the cluster, SRC) were acquired and visually inspected. Molecular mechanics-generalized born surface area (MM-GBSA) calculations were performed on 100 frames belonging to the most populated cluster of PCSK9/peptide conformations, and the MMPBSA.py module [33] of AMBER20 was used to this aim, keeping parameters in the default values. In these calculations, the single trajectory approach was applied and the entropy contributions to the binding free energy was neglected [25,34]. For this reason, the estimated binding free energy values are termed by us ΔG*.

### 2.3. Alanine Scanning

The nine P5 alanine mutants were built systematically altering the peptide sequence on the PCSK9/P5 complex, by the tleap module of AMBER20 [28]. The resulting complexes were energy minimized and equilibrated by accomplishing 250 ns of MD simulations, adopting the procedure and the parameters previously described for the PCSK9/P5 complex. A hundred of snapshots were regularly extracted from the trajectory frames in which the peptide under investigation displayed the smallest root mean square deviation (RMSD) value fluctuation, to ensure the lowest standard error in the binding free energy calculation. MM-GBSA calculations by MMPBSA.py module [33] were finally performed to estimate the binding free energy values of the mutant peptides.

### 2.4. Computational Design of New P5 Analogs

The “protein preparation wizard” module implemented in the Maestro release 2019-4 (Schrödinger, LLC, New York, NY, USA, 2017) for molecular modeling ensured the accuracy of the PCSK9/P5 complex conformation previously equilibrated by MD simulations. In particular, this module permitted: (1) to check the residue protonation state at pH 7.4, (2) to check the residue completeness, (3) to eliminate atomic clashes, and (4) to assign the OPLS3e force field [35]. Then, the 400 possible peptides were generated by the replacement of the P5 positions 2 and 9 with the twenty natural amino acids. The resulting PCSK9/peptide complexes were minimized by Prime MM-GBSA module of Maestro, which uses OPLS3e [35] as force field and a continuum solvent models to include the solvent effect into the calculations. Then, affinity maturation functionality implemented in Bioluminate module (Schrödinger, LLC, New York, NY, USA, 2017) estimated the change in affinity (ΔAffinity) between PCSK9 and the mutant peptides, with respect to P5. Finally, the mutant peptides acquiring the highest gain in ΔAffinity were additionally refined by MD simulations and MM-GBSA calculations by AMBER20 package [28], as it was conducted previously for the alanine-mutant peptides (see the previous section for details).

### 2.5. HMG-CoA Reductase Model Setup and Simulations Protocol

The HMG-CoAR structure solved by X-ray crystallography (PDB accession code 3CCZ) [36] used in this study was deposited as a homotetramer in which the (3R,5R)-7-[2-(4-fluorophenyl)-4-{[(1S)-2-hydroxy-1-phenylethyl]carbamoyl}-5-(1-methylethyl)-1H-imidazol-1-yl]-3,5-dihydroxyheptanoic acids are bound in the catalytic sites. For simplicity, we have performed simulations on the functionally active homodimer, choosing as peptide binding site the one identified by the presence of a statin in the X-ray complex. Since the homodimer contained two statin molecules, one of them was removed to allow the docking calculations, while the statin present in the second site was kept in its original position, to avoid any protein conformational distortion inducted by the absence of the ligand. The system was prepared and minimized through the “protein preparation wizard” tool available in the Maestro software (release 2019-4). Peptide docking calculations of [S7A]P5 were performed by using the Glide docking tool [37] of Maestro software, setting as center of the grid the centroid of the statin (residue code 5HI), co-crystallized in the catalytic site of the HMG-CoAR. The “standard precision” mode was applied in these calculations, and the Glide gscore was applied as scoring algorithm. Ten peptides’ docking poses were generated, while the number of post-docking minimization poses was set to 50. The formation of *cis* amide bonds was not allowed. The [S7A]P5 pose with the best-predicted gscore (−9.881 kcal/mol) was selected for the following MD simulations and cluster analysis calculations (Table S1, Supporting Material), adopting the AMBER20 protocol [28] previously described for the studies of the peptides in complex with PCSK9.

### 2.6. Peptide Synthesis

The Genscript (Piscataway, NJ, USA) synthesized for us the P5 analogs selected for biological assays. All compounds are >95% pure by HPLC analysis (see Supporting Information for details).

### 2.7. HepG2 Cell Culture Conditions and Treatment

The HepG2 cell line was bought from ATCC (HB-8065, ATCC from LGC Standards, Milan, Italy) and was cultured in DMEM high glucose with stable L-glutamine, supplemented with 10% FBS, 100 U/mL penicillin, and 100 µg/mL streptomycin (complete growth medium) with incubation at 37 °C under 5% CO_2_ atmosphere.

### 2.8. HMG-CoAR Activity Assay

The experiments were carried out following the manufacturer instructions and optimized protocol [38]. See Supplementary Materials for further details.

### 2.9. In Vitro PCSK9-LDLR Binding Assay

Peptides P5 and P5 analogs (0.1–100 µM) were tested using the in vitro PCSK9-LDLR binding assay (CycLex Co., Nagano, Japan) following the manufacture instructions and conditions already optimized [21]. Further details are provided in Supplementary Materials.

### 2.10. In-Cell Western (ICW) Assay

For the experiments, a total of 3 × 10^4^ HepG2 cells/well were seeded in 96-well plates and treated with 4.0 μg/mL PCSK9-WT and 4.0 μg/mL PCSK9 + peptides P5 and/or P5 analogs (50 µM) and vehicle (H_2_O) for 2 h at 37 °C under 5% CO_2_ atmosphere. Thus, cells underwent to ICW assay following conditions already optimized [26]. See Supplementary Materials for detailed information.

### 2.11. Fluorescent LDL Uptake

HepG2 cells (3 × 10^4^/well) were seeded in 96-well plates and kept in complete growth medium for 2 days before treatment. The third day, cells were washed with PBS and then starved overnight (O/N) in DMEM without FBS and antibiotics. After starvation, they were treated with 4.0 μg/mL PCSK9 and 4.0 μg/mL PCSK9 + P5 and P5 analogs peptides (50 µM), and vehicle (H_2_O) for 2 h with at 37 °C under 5% CO_2_ atmosphere. Fluorescent LDL-uptake was finally assessed following optimized protocol [22]. See Supplementary Materials for further details.

### 2.12. Western Blot Analysis

A total of 1.5 × 10^5^ HepG2 cells/well (24-well plate) were treated with 50 μM of P5 and P5 analogs for 24 h. Immunoblotting experiments were performed using optimized protocol [39]. See Supplementary Materials for further details.

### 2.13. Quantification of PCSK9 Secreted by HepG2 Cells through ELISA

The supernatants collected from treated HepG2 cells (50 μM of P5 and/or P5 analogs) were centrifuged at 600× *g* for 10 min at 4 °C and ELISA assay performed using protocol already optimized [40]. Detailed data are provided in Supplementary Materials.

### 2.14. Statistical Analysis

All the data set were checked for normal distribution by D’Agostino and Pearson test. Since they are all normally disturbed with *p*-values < 0.05, we proceeded with statistical analyses by one-way ANOVA followed by Tukey’s post-hoc tests and using Graphpad Prism 9. Values were reported as means ± S.D.; *p*-values < 0.05 were considered significant.

## 3. Results and Discussion

### 3.1. Identification of Hotspots and Designs of New P5 Analogs

**Figure 1:**
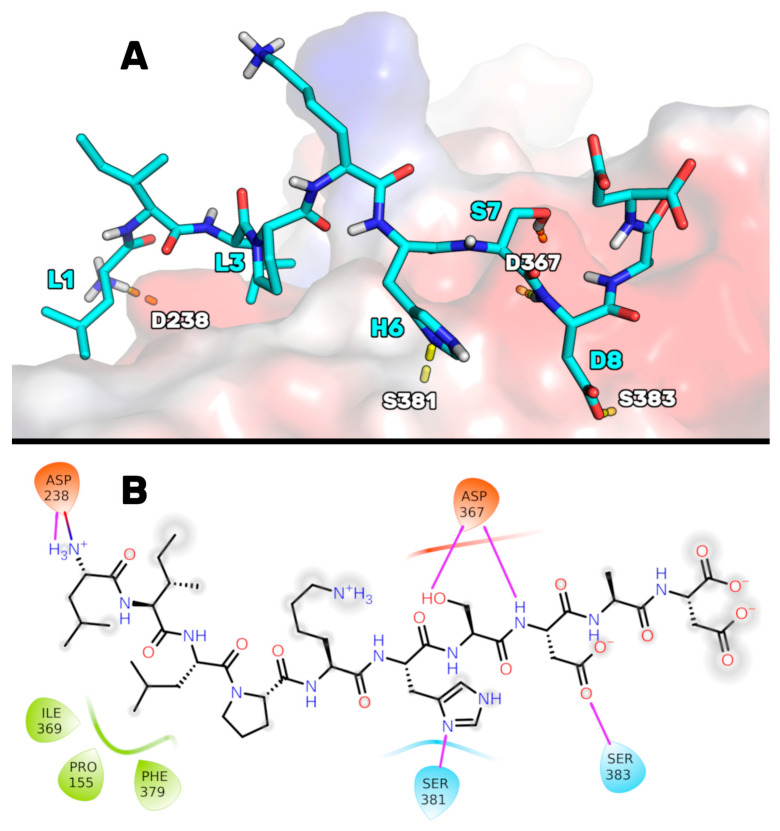
(**A**) The expected P5 binding mode on the PCSK9 surface after additional MD simulations. Yellow dotted lines highlight the H-bond network. The enzyme solvent-accessible surface is depicted accordingly by the partial charge of the residues: blue for positive areas and red for negative areas. Peptide P5 is represented as cyan sticks. (**B**) 2D representation of the predicted P5 binding mode in complex with PCSK9. H-bonds and salt bridges are highlighted in purple and blue/red lines, respectively. Hydrophobic residues interacting with P5 are colored in green.

To obtain a robust hypothesis on the peptide P5 binding mode, the PCSK9/P5 complex model we had previously developed [21] was optimized once more by extending the molecular dynamics (MD) simulations to 1 µs (see Figure S1 in the Supporting Information for the RMSD plots). At the end of these simulations, the MD trajectory frames were grouped using a cluster analysis algorithm (see the Materials and Methods Section) to determine which was the most favored P5 conformation in complex with PCSK9. The PCSK9/P5 complex conformation representative of the most populated cluster (78%) suggested that peptide P5 could bind to PCSK9, as illustrated in Figure 1. In particular, P5 could bind to PCSK9 through (1) a salt bridge between the charged NH term of P5-Leu1 and the side chain of Asp238, (2) an H-bond between the imidazole ring of the P5-His6 and the NH group of Ser381, (3) an H-bond network between the side chain of P5-Ser7, the NH of P5-Asp8, and the side chain of Asp367, and (4) an H-bond shaped by the side chain of P5-Asp8 and the side chain of Ser383. The side chain of P5-Leu3 was deeply inserted into a hydrophobic basin sized by the PCSK9 residues Phe379, Pro155, and Ile369, creating van der Waals interactions.

Then, to identify new P5 analogs endowed with improved PCSK9 affinity, we designed new peptides by substituting the P5 residues not considerably involved in the PCSK9 contact (non-hotspot) with new amino acids showing an improved complementarity with the PCSK9 surface [25]. In the first step, the hotspots and non-hotspots of P5 were discovered by performing a computational alanine-scanning mutagenesis analysis, in which all the peptide residues in the PCSK9/P5 complex were systematically mutated into alanine. Specifically, nine 3D models, in which PCSK9 was in complex with each alanine-mutated peptide P5, were simulated by 200 ns-long MD simulations, and the subsequent molecular mechanics-generalized born surface area (MM-GBSA) calculations estimated the mutant peptides’ binding free energy values (ΔG*, Table 1). Then, by comparing the ΔG* values calculated for the mutant peptides with those calculated for P5, identifying the hotspots and non-hotspots of P5 was possible [40,41].

The attained results suggest that positions 3 and 6 can be considered hotspots, as their mutation into alanine led to P5 analogs endowed with a considerable reduction of the predicted binding affinity (ΔΔG* higher than 10 kcal/mol). Specifically, P5-His6 seemed crucial for peptide binding because its substitution led to a dramatic drop in the peptide binding interaction energy. MD simulations suggest that the substitution of the basic side chain of His6 with a methyl group led to a change in the peptide binding mode due to a lack of an H-bond between the PCSK9-Ser282 amide group and the imidazole ring of P5-His6. For this reason, the [H6A] peptide P5 was unbound from the PCSK9 surface after the initial steps of the MD simulations (see Figure S2 in the Supporting Information for the RMSD plots). Similarly, the removal of the side chain of P5-Leu3 led to a peptide incapable of maintaining the P5 initial binding mode, as the hydrophobic contacts engaged by the Leu3 isobutyl group with the hydrophobic crevice sized by the PCSK9 residues Leu159, Pro156, Ala240, and Ile370 were missing.

The alanine mutation of Leu1 and Lys5 led to peptides with a calculated binding affinity that was slightly lower than that of P5 (ΔΔG* close +5 kcal/mol). However, given the inaccuracy of the MM-GBSA calculations and the observation that the side chains of Leu1 and Lys5 fluctuating in a solvent environment do not stably bind to PCSK9 during the MD simulations, positions 1 and 5 cannot be considered strong hotspots similar to positions 3 and 6. Conversely, the substitution with alanine of Ile2, Ser7, and Asp10 of P5 led to peptides with a predicted binding affinity close to that predicted for the template peptide. Therefore, they can be considered non-hotspots and can potentially be substituted with different amino acids. However, the predicted data on Leu1 and Ile2 are in accordance with our recent experimental data, which show that a metabolite of peptide P5 that does not contain the first two residues (P5-met, LPKHSDAD) displays an IC_50_ value close to the parent peptide P5 [24].

Conversely, the P4A and D8A mutant peptides showed a higher affinity to PCSK9 than P5. However, as the gain in the ΔG* value was not extremely high, the synthesis and biological evaluation of these peptides was not considered suitable.

### 3.2. Design of P5 Analogs with Improved PCSK9 Predicted Affinity

The alanine-scanning study showed that the positions 1, 2, 7, and 10 on the P5 sequence could be considered non-hotspots. Moreover, the alanine in position 9 should be considered a non-hotspot, as alanine is already present in the natural P5 sequence. Nevertheless, the P5-Ser7 OH group could create an H-bond with PCSK9-Asp367, and the P5-Asp10 side chain could be involved in the fold of the peptide, as an internal H-bond could be shaped with the side chain of P5-Ser7. Thus, we decided to mutate the residues in positions 2 and 9 to develop novel P5 analogs with improved PCSK9 binding affinity.

Accordingly, with this assumption, 20^2^ peptide P5 analogs were computationally designed through the systematic substitution of positions 2 and 9 with all natural amino acids. Their theoretical affinity for PCSK9 was preliminary evaluated by the Prime algorithm (Maestro, release 2019-4), which can estimate the peptide binding free energy using the MM-GBSA approach. PCSK9 in complex with the 10 top-ranking P5 analogs (i.e., those with the lowest ΔAffinity values, Table 2) again underwent MD simulations by applying the previously described AMBER20 MD protocol. The ΔG* values were estimated using the MM-GBSA protocol (Table 2), which allowed for the acquisition of ΔG* values comparable with those previously attained for P5 and other P5 alanine mutants.

**Figure 2:**
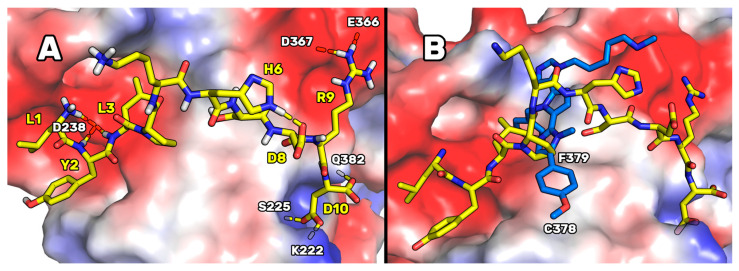
(**A**) Expected binding mode of [I2Y-A9R]P5 (i.e., P5-Best) on the PCSK9 surface resulting from the MD simulations and cluster analysis. Yellow dotted lines depict the H-bond network. The enzyme solvent-accessible surface is colored according to the partial charge of the residues: blue for positive areas and red for negative areas. P5-Best is represented as yellow sticks. (**B**) Superimposition of P5-Best and Rim13, both bound on the PCSK9 surface.

The Prime calculations (third column of Table 2) suggested that the peptides acquiring an improved predicted binding energy were the ones containing arginine in position 9. At variance, the substitutions in position 2 did not considerably affect the affinity of the resulting peptides (differences in the ΔAffinity values followed in the range of 5 kcal/mol). Subsequently, using the AMBER20/MM-GBSA calculations, the resulting ΔG* values spanned from −19 to −42 kcal/mol. This allowed us to assess that the peptide [I2Y-A9R]P5 (i.e., P5-Best) was endowed with the highest predicted PCSK9 binding affinity (see Figure S3 in the Supporting Information for the RMSD plots). In fact, the ΔG* value of P5-Best was two times the value predicted for the template peptide P5, suggesting that P5-Best could show an affinity to PCSK9 appreciably lower than P5. Our simulations showed that, as indicated in the conformation representative of the most populated cluster (Figure 2), P5-Best could acquire an ameliorated PCSK9 complementarity because of the possibility of creating two salt bridges: the first between the new arginine in position 9 and the side chains of Glu366 and Asp367, and the second between the side chain of P5-Best-Asp10 and the side chain of Lys222. These interactions were also enforced by the presence of an H-bond between the P5-Best-Asp10 and the OH group of Ser225. Moreover, the P5-Best-Ile3 side chain was in contact with the hydrophobic pocket sized by Phe379, while the phenol ring of the new residue P5-Best-Tyr2 was located close to Arg194. The NH groups of P5-Best-Tyr2 and -Ile3 created two H-bonds with the side chain of Asp238.

These results were also compared to the computational data attained designing the poly-imidazole derivatives capable of inhibiting PCSK9 [17]. In fact, in our previous paper, by applying a computational approach such as the one here applied, we designed and biologically evaluated two poly-imidazole derivatives endowed with PCSK9 inhibiting activity. The biological evaluation of the most interesting poly-imidazoles, named Rim13 and Rim14, allowed us to report on their ability to modulate the LDLR expression on the human hepatic HepG2 cell surface, and their capacity to increase the extracellular uptake of LDL by the same cells. Here, structurally aligning the P5-Best and the Rim13 hypothetical binding modes, we noted that the backbone atoms of the peptide residues Pro4 and Lys5 were mimicked by the first two imidazole rings of Rim13 (Figure 2B). Moreover, the benzyl chain of the second imidazole ring of Rim13 was projected in the same hydrophobic cleft shaped by Phe379 and occupied by the side chain of P5-Best-Leu3, creating van der Waals interactions. Furthermore, the negatively charged area created by the PCSK9 residues Asp367 and Glu366 were in contact with the side chain of the P5-Best-Arg9 and the amino-methyl chain of Rim13. Since they bind similarly, creating contacts with the same PCSK9 residues, this alignment could help in the design of new poly-imidazole derivatives. In fact, aiming at designing more potent poly-imidazoles derivatives, the benzyl moiety of Rim13 could be substituted by alkyl chains (linear or not), to reproduce the interactions played by the P5-Best-Leu3 residue. Conversely, regarding the design of new P5 analogs, the Pro4 of P5-Best could be replaced by aromatic residues such as Phe, Tyr or Trp, in order to reproduce the interactions played by the *p*-methoxyphenyl ring of Rim13. However, the oral PK properties of peptides remains strongly limited by the presence of degrading enzymes in the gastrointestinal tract. Nevertheless, the research efforts are still devoted to solve this limitation. In fact, active peptides could be orally administered together with penetration enhancers, within hydrogels or in combination with digestive enzyme inhibitors. As alternative, they can be suitably coated by acid-stable polymers or administered through intestinal patches [42]. By means of one of these innovative delivery strategies, even peptides active in the high micromolar range could be successfully employed for the treatment of several pathologies. Actually, numerous peptides are in phase III of clinical trials but, until now, only desmopressin is available in the market, and used in the clinic [42].

### 3.3. Experimental Validation of the Computational Predictions

In light of these theoretical studies, empirical assays were performed on the [H6A] peptide P5 (i.e., P5-H6A) because position 6 was recognized as a hotspot (Table 1), on P5-Best because of its lowest predicted binding free energy value, and on [S7A]P5 (i.e., P5-S7A) because it represents one of the peptides for which the alanine mutation did not remarkably alter the predicted binding free energy value. Conversely, the mutation of P5-Asp10 into alanine affected peptide folding (as shown by MD simulations) and the water solubility of the peptide, as the negatively charged side chain of Asp10 should be substituted with the aliphatic methyl group of alanine. Thus, the peptides P5-H6A, P5-S7A, and P5-Best were purchased by GenScript and biochemically evaluated by in vitro experiments.

### 3.4. P5 Analogs Impair the PPI between PCSK9 and LDLR

**Figure 3:**
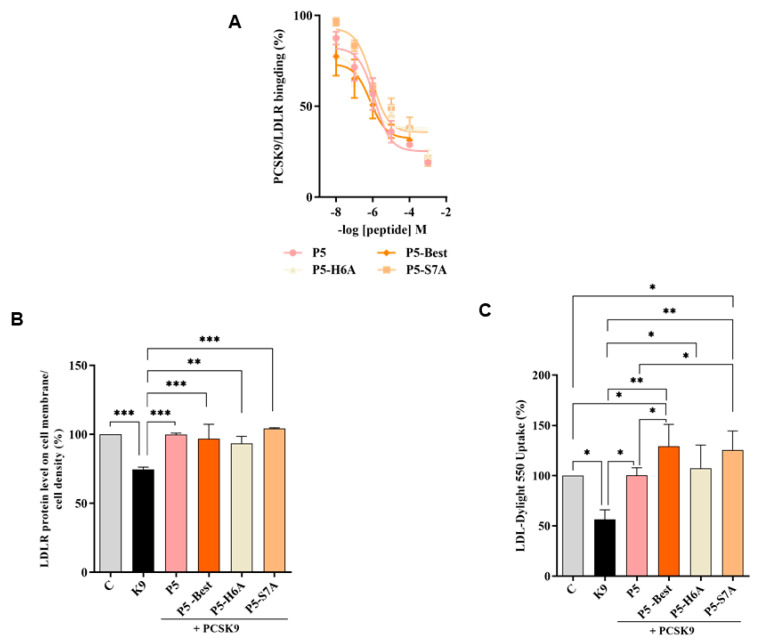
Inhibition of the PPI between PCSK9 and LDLR. (**A**) Impairment of the protein-protein interaction between PCSK9 and LDLR. (**B**) The treatment of HepG2 cells with PCSK9 (K9 in the graphs, 4 µg/mL) reduced the active LDLR protein levels localized on the surface of the cells, which were restored by P5 and P5 analogs (50 µM). (**C**) The decreased functional ability of HepG2 cells to absorb LDL from the extracellular space observed after incubation with PCSK9 (4 µg/mL) improved after treatment with P5 and P5 analogs (50 µM). The data points represent the average ± SD of four independent experiments performed in duplicate. Data were analyzed using one-way ANOVA, followed by Tukey’s post-hoc test; (*) *p* < 0.05, (**) *p* < 0.01, and (***) *p* < 0.001. C: control sample.

To verify whether the P5 derivatives could impair the PPI between PCSK9 and LDLR, dedicated biochemical experiments were assessed. The results showed that P5-Best, P5-H6A and P5-S7A reduced the PCSK9-LDLR binding with a dose-response trend and IC_50_ values of 0.7, 9.0, and 1.45 μM, respectively (Figure 3A). The results confirmed that two of the new P5 derivatives were more active than P5 (1.6 μM). These data are in line with the computational predictions. In fact, the peptides ∆G* value calculations indicated that the most active peptide could be the double mutant peptide P5-Best (∆G= −41.7 kcal/mol) while, among other ones, P5-S7A should display binding affinity in the range of P5 (∆G* values of −19.3 and −18.9 kcal/mol, respectively), and P5-H6A should be do not active, since by our predictions, the side chain of H6 plays a crucial role in the stabilization of the peptide on the PCSK9 surface. Nevertheless, it has to be stated that a higher affinity should be expected for P5-Best, since the calculated ∆G* value calculated for this peptide was double than that of P5. In our opinion, the lack of linearly between the binding affinity experimental data (IC_50_ values) and the computational predictions, could be due to the omitted calculation of the entropic contribution to binding free energy value. In fact, the calculation of this contribution is highly computationally demanding, and the error associated with the estimation is very often greater than the value itself. Moreover, the data attained by the further biological investigations on these peptides cannot be compared with the computational predictions, since it is very difficult to discuss the in silico results in comparison with the biological data obtained from the HepG2 cells. In fact, the molecular modeling studies have been performed on a PCSK9 model immersed in a box of water molecules and the biological experiment capable of reproducing these conditions is only the one in which the recombinant PCSK9 is in contact with the LDLR, i.e., the binding assays displayed in Figure 3. Conversely, when the biological properties of peptides are assessed in complex experimental conditions, such as the one in which cells are involved, the molecular modeling results cannot be linearly compared with the experimental data. In fact, the effects of membranes, extracellular or intracellular enzymes cannot be considered by our calculations.

Furthermore, the ability of these P5 analogs to modulate the levels of LDLR localized on HepG2 surfaces was investigated in the presence of PCSK9 (4 μg/mL) using an in-cell western (ICW) assay. The results showed that the LDLR levels decreased in the presence of PCSK9 alone by 25.4 ± 1.6% (*p* < 0.001) compared with the untreated control cells, and that P5-Best, P5-H6A, and P5-S7A could significantly restore the LDLR levels to 96.9 ± 10.4%, 93.4 ± 5.2%, and 104.4 ± 0.4% (*p* < 0.001), respectively, when co-incubated with PCSK9 (Figure 3B). Finally, functional cell-based assays were performed to investigate the ability of HepG2 cells to uptake extracellular LDL in the presence of PCSK9. HepG2 cells incubated with PCSK9 alone showed a 43.6 ± 9.6% (*p* < 0.05) reduction in the uptake of fluorescent LDL compared with the untreated control cells. This result is in agreement with the reduction of active LDLR population on the cell surface, which was observed by ICW. After co-incubation with PCSK9, P5-Best, P5-H6A, and P5-S7A completely restored the LDLR function, increasing the LDL uptake to 129.2 ± 21.9% (*p* < 0.001), 107.4 ± 23.0% (*p* < 0.05), and 125.4 ± 19.0% (*p* < 0.01) (Figure 3C), respectively.

P5 analogs demonstrated to be more active than peptide Pep2-8 (TVFTSWEEYLDWV) [43] and its analogs [Y9A]Pep2-8 and [T4R,W12Y]Pep2-8 [44] as PPI inhibitors of PCSK9. More in details, at the fixed concentration of 100 µM, Pep2-8 impaired the PCSK9-LDLR binding by −36.5% vs. the control, whereas [Y9A]Pep2-8 and [T4R,W12Y]Pep2-8 by −69.8% and −93.0%, respectively [44]. Indeed, the IC_50_ value of [Y9A]Pep2-8 was equal to 27.12 ± 1.2 µM and that of [T4R,W12Y]Pep2-8 equal to 14.50 ± 1.3 µM [44], clearly indicating that P5-Best is about 30- and 20-fold more potent than the Pep2-8 mutant peptides. In addition, at cellular levels, Pep2-8 and both Pep2-8 analogs were less efficient than P5 analogs to restore the LDLR protein levels and the functional ability of hepatocytes to absorb LDL from the extracellular environment [44]. On the contrary, P9-38, a cyclized Pep2-8 analogue, demonstrated to be 35-fold more potent than P5-Best in impairing the PPI between PCSK9/LDLR displaying and IC_50_ equals to 20 nM and it was 1000-fold more potent to restore the LDLR level and functionality in HepG2 cells [45].

Finally, P5-Best is slightly more potent than the poly-imidazole Rim13 which inhibit the interaction between PCSK9 and LDLR by an IC_50_ equals to 1.4 µM, a value similar to the reference peptide P5. In the same range of concentration of Rim13, P5 analogs successes in the restoring the functional activity of LDLRs on the surface of hepatocytes preventing their degradation [45].

Although all P5 analogs successfully restored the level of LDLR protein similar to peptide P5, statistical analysis revealed that from a functional point of view, both P5-Best and P5-S7A not only restored the ability of hepatic cells to uptake LDL from the extracellular environment but also improved this capability against untreated cells. These results suggest that the hypocholesterolemic effect occurs with a dual mechanism of action involving the modulation of HMG-CoAR activity and protein levels. To assess this aspect and deepen this behavior, further HMG-CoAR activity assay and western blot experiments were performed.

### 3.5. P5 Analogs Modulate the Hepatic LDLR Pathway by Inhibiting HMG-CoAR Activity

**Figure 4:**
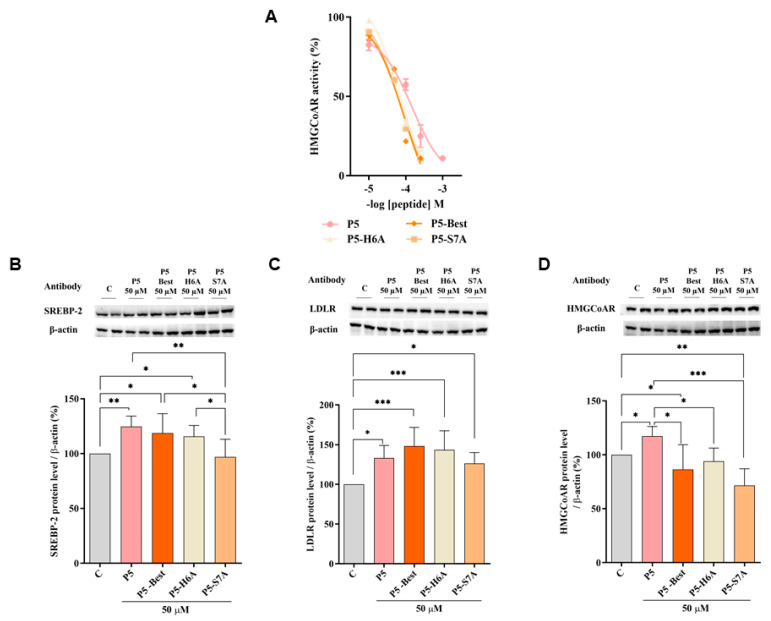
Modulation of the LDLR pathway in HepG2 cells treated with P5 and P5 analogs. (**A**) In vitro inhibition of HMG-CoAR activity. (**B**) The effect on SREBP-2 protein levels, (**C**) LDLR protein levels, and (**D**) HMG-CoAR protein levels after the treatment of HepG2 cells with P5 and P5 analogs, respectively. Data points represent the average ± SD of four independent experiments performed in duplicate. C vs. P5 and P5 analog samples were analyzed using a one-way ANOVA, followed by by Tukey’s test; (*) *p* < 0.05, (**) *p* < 0.01, and (***) *p* < 0.001. C: control sample.

To better characterize the dual inhibitory activity of all P5 analogs, a biochemical investigation was conducted to assess their effect on the modulation of HMG-CoAR activity. The results suggested that P5-Best, P5-H6A, and P5-S7A inhibited enzyme activity with an IC_50_ of 88.9, 74.4, and 73.8 µM, respectively, showing more effective inhibitory activity than P5 (147.2 µM) (Figure 4A), but they are still less active than statins. In facts, the IC_50_ values for the inhibition of HMG-CoAR activity for pravastatin simvastatin, atorvastatin, and rosuvastatin are equals to 44.1, 11.2, 8.2, and 5.4 nM, respectively [46,47].

Even though, P5 and P5 analogs are less active than statins as HMG-CoAR inhbitors and their clinical implication is still too far, they display the unique feature to inhibit both HMG-CoAR and PCSK9 targets, making them lead compounds for developing new peptidomimetic and/or small molecules endorsed by improved activity on both targets involved in the control of the circulating cholesterol level.

Further experiments were performed to verify the ability of these P5 analogs to modulate the LDLR pathway in HepG2 cells. Similar to P5, P5-Best and P5-H6A induced an upregulation of the SREBP-2 transcription factor level up to 118.6 ± 17.7% and 115.6 ± 10.1% (*p* < 0.05) (Figure 4B), respectively, resulting in an augmentation of the LDLR protein levels up to 148.4 ± 23.4% and 143.5 ± 24.0% (*p* < 0.001), respectively (Figure 4C). Interestingly, although P5-S7A caused a slight reduction of the SREBP-2 protein level to 96.9 ± 16.1% (Figure 4B), it led to an increase in the LDLR protein level up to 126.5 ± 13.6% (*p* < 0.05) (Figure 4C). Thus, in contrast to P5, P5-Best, and P5-H6A, the upregulation of the LDLR protein level and activity induced by P5-S7A was not through SREBP-2 pathway activation. Notably, P3 (YDFYPSSTKDQQS), a peptide from lupin protein that inhibits HMG-CoAR activity, leads to an increase in the LDLR protein levels without SREBP-2 activation but through the compensatory upregulation of the SREBP-1 [48]. Therefore, it was hypothesized that P5-S7A could possess the same effect as P3 on LDLR protein production through the regulation of the SREBP-1 pathway. However, unlike P5, P5-Best, P5-H6A, and P5-S7A decreased the HMG-CoAR levels up to 86.5 ± 22.8% (*p* < 0.05), 94.1 ± 12.2%, and 71.6 ± 15.5% (*p* < 0.01), respectively (Figure 4D), indicating that the P5 analogs were more active as HMG-CoAR inhibitors than P5. Interestingly, P5-Best, P5-H6A, and P5-S7A which are about two-fold more potent than P5 as both HMG-CoAR and PCSK9/LDLR PPI inhibitors, respectively, are also more efficient in the reduction of the HMG-CoAR protein levels with a direct effect in the intracellular cholesterol homeostasis. Indeed, overall P5-Best and P5-S7A can improve the functional ability of hepatic cells to absorb extracellular LDL (Figure 4D).

### 3.6. P5 Analogs Modulate the Hepatic PCSK9 Pathway

**Figure 5:**
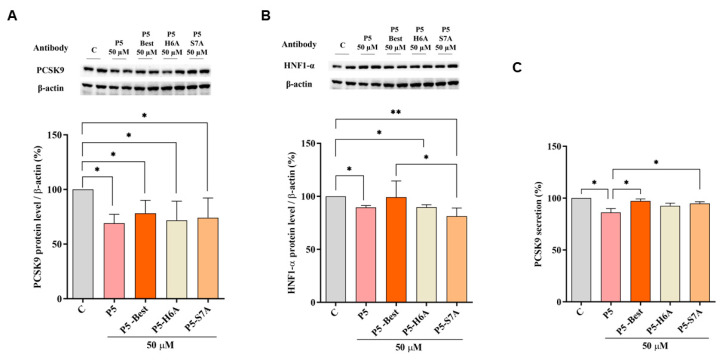
Modulation of the PCSK9 pathway in HepG2 cells. (**A**) Effects on the PCSK9 protein levels, (**B**) effects on the HNF1-α protein level, and (**C**) effects on mature PCSK9 secretion. Data points represent the average ± SD of six independent experiments performed in duplicate. C vs. P5 and P5 analog samples were analyzed using a one-way ANOVA, followed by by Tukey’s test; (*) *p* < 0.05 and (**) *p* < 0.001. C: control sample.

Figure 5 shows that P5-Best, P5-H6A, and P5-S7A decreased the PCSK9 protein levels by 21.8 ± 11.8%, 28.2 ± 17.5%, and 25.8 ± 17.9%, respectively (*p* < 0.05) (Figure 5A). Moreover, also the HNF1-α protein levels were decreased by 1.2 ± 15.4%, 10.3 ± 2.4% (*p* < 0.05), and 18.7 ± 7.6% (*p* < 0.01) (Figure 5B), respectively.

Although the ability to reduce the secretion of mature PCSK9 was weaker than that of P5, P5-Best, P5-H6A, and P5-S7A could also induce a slight reduction by 2.7 ± 1.9%, 7.4 ± 2.6%, and 5.1 ± 1.7%, respectively (Figure 5C). These results agree with the behavior of another naturally peptide from soybean β-Conglycinin [49]. In particular, YVVNPDNNEN peptide (at 250 µM) reduces the PCSK9 protein level and its secretion via the modulation of HNF1-α [49]. Our results suggest that lupin P5 and its new analogs, being active at 50 µM in the hepatic cells, are 5-fold more potent than YVVNPDNNEN. In addition, it was also demonstrated that YVVNPDNNEN it is not a dual inhibitor peptide since being able to inhibit the HMG-CoAR activity [50] but not the PPI between PCSK9 and the LDLR [49].

### 3.7. P5 Analogs Increase the Expression of LDLR Localized in the Cellular Membranes and Modulate LDL Uptake in HepG2 Cells

**Figure 6:**
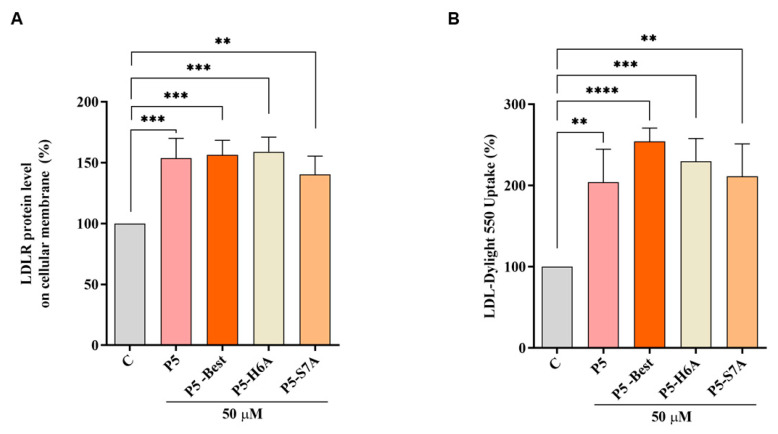
Modulation of the LDLR pathway in HepG2 cells. (**A**) The effect on the LDLR localized on the surface of HepG2 cells after the treatment of HepG2 cells with P5 and P5 analogs, respectively. (**B**) Enhancement of the functional ability of HepG2 cells to uptake LDL from the extracellular environment. Data points represent the average ± SD of four independent experiments performed in duplicate. C vs. P5, and P5 analog samples were analyzed using one-way ANOVA, followed by by Tukey’s test; (**) *p* < 0.01, (***) *p* < 0.001, and (****) *p* < 0.0001. C: control sample.

In accordance with the above results, P5-Best, P5-H6A, and P5-S7A increased the LDLR levels localized in the cellular membranes of HepG2 cells by 156.3 ± 12.1%, 158.9 ± 12.0% (*p* < 0.001), and 140.2 ± 15.1% (*p* < 0.01) at 50 µM, respectively (Figure 6A). Experiments were also performed in parallel, with P5 as the reference compound, which increased the membrane LDLR protein levels by 153.6 ± 16.4% (*p* < 0.001) at the same concentration of 50 µM. Consequently, the functional capability of HepG2 cells to uptake extracellular LDL after treatments with P5, P5-Best, P5-H6A, and P5-S7A was observed, leading to an increased ability of 203.8 ± 40.67% (*p* < 0.01), 254.3 ± 16.4% (*p* < 0.0001), 229.8 ± 27.9% (*p* < 0.001), and 211.1 ± 40.1% (*p* < 0.01), respectively (Figure 6B).

### 3.8. Docking of P5-S7A and MD Simulations on HMG-CoAR

**Figure 7:**
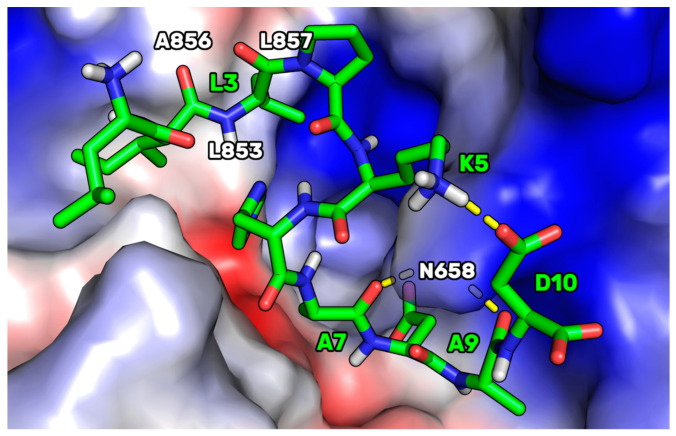
The representative structure of the most populated cluster of P5-S7A (green sticks) bound to HMG-CoAR (electrostatic surface). The small hydrophobic pocket (Leu853, Ala856, and Leu857) interacting with P5-S7A-Leu3 is highlighted. The H-bonds are represented by yellow dashed lines. Only polar hydrogens are shown in the figure.

The experimental assays on the purchased peptides highlighted the improvement in the dual inhibitory activity of the P5 mutant peptides. Specifically, P5-S7A showed the lowest IC_50_ value for HMG-CoAR. Thus, docking and MD simulations were conducted to acquire atomistic details on the putative binding mode of P5-S7A in complex with HMG-CoAR. This study can pave the way for the design of more dual-active peptides. P5-S7A was docked to the statin binding site of HMG-CoAR using Glide (see the Experimental Section for details), and the best docking pose (gscore = −9.881 kcal/mol) was selected for further 500 ns-long MD simulations in explicit water solvent (see Figure S4 in the Supporting Information for the RMSD plots). As the enzyme was in the dimeric state, the statin present in the other binding sites was not deleted (see the Experimental Section for details) to preserve the overall folding of the simulating system. At the end of the MD simulations, the root mean square deviation (RMSD) of the peptide was analyzed, and the peptide conformations sampled during the MD production run were clustered using the average-linkage method, which was previously prescribed for the PCSK9/peptide complexes. The results showed that only one cluster was mainly populated, representing 73.1% of the peptide conformations. The structure representative of this cluster is depicted in Figure 7.

This HMGCoAR/P5-S7A complex showed the presence of a H-bond network between the P5-S7A-Ala7 and -Ala9 backbone atoms, with a side chain of HMG-CoAR-Asn658. P5-S7A-Leu3 projected its side chain in a small hydrophobic pocket sized by HMGCoAR-Leu853, -Ala856, and -Leu857. Interestingly, the presence of an intramolecular H-bond between the side chains of P5-S7A-Lys5 and -Asp10 improved the overall peptide conformational stability. Moreover, the supposed binding mode of P5-S7A was consistent with the binding affinity data, indicating that the IC_50_ of P5-H6A on HMG-CoAR was close to that of P5-S7A. Both residues could point their side chains to an effectively empty pocket sized by HMG-CoAR-Leu853, -Ala856, and -Leu857, which did not create any interactions with the HMG-CoAR counterpart. Thus, the substitution of positions 6 and 7 with alanine did not elicit any strong variation in the experimental binding affinity. This hypothesis paves the way for the design of new P5 analogs in which positions 6 and 7 can be mutated by unnatural amino acids capable of creating stronger interactions with HMG-CoAR.

**Figure 8:**
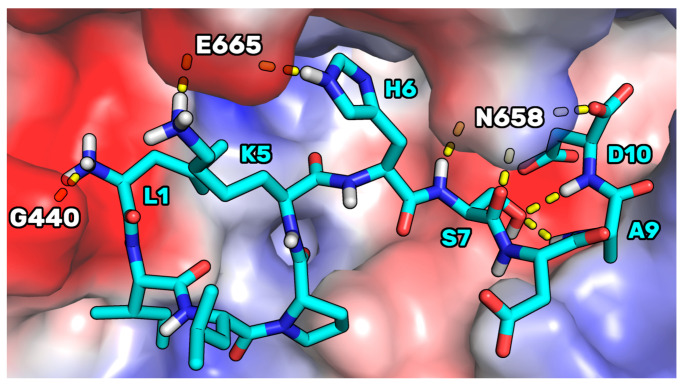
The representative structure of the most populated cluster of P5 (cyan sticks) bound to HMG-CoAR (electrostatic surface). H-bonds are represented by yellow dashed lines. Only polar hydrogens are shown in the figure.

The binding mode supposed for P5-S7A was then compared to the one of P5 in complex with HMG-CoAR, to understand the possible reasons on the base of the improved binding affinity displayed by the mutant peptide. In our previous article [21] we have reported on the results of docking calculations of P5. Here, performing MD simulations starting from the P5 docking pose (see Figure S5 in the Supporting Information for the RMSD plots), we noted that, in the complex conformation representative of the most populated cluster (70%), P5 adopted a binding mode in which the side chain of P5-S7 created two intramolecular H-bonds with the NH groups of P5-A9 and P5-D10 (Figure 8). In the mutant peptide P5-S7A, these internal bonds cannot be created for the absence of the OH group in position 7. This, in our opinion, led to a peptide endowed with an increased conformational freedom, leaving the C-terminal residues to adopt a cyclic conformation in which an internal salt bridge can be shaped between the side chains of P5-K5 and P5-D10. This conformation could be more prone to create remodeled and ameliorated interactions with the enzyme.

**Figure 9:**
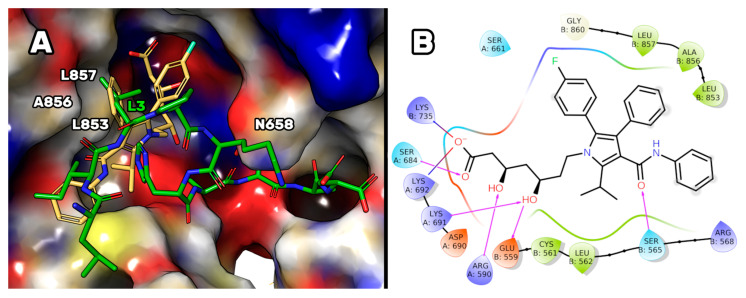
(**A**) Crystallographic pose of atorvastatin (yellow sticks), as was found in the X-ray structure available in the PDB (accession code 1HWK [51]), superimposed on the representative structure of the most populated cluster of P5-S7A (green sticks) in complex with HMG-CoAR. (**B**) 2D representation of the protein-ligand contacts displayed by atorvastatin (cutoff = 3.00 Å) in complex with HMG-CoA, as in the X-ray structure. H-bonds are highlighted by purple arrows and the hydrophobic residues involved in the protein-ligand interactions are colored in green.

Finally, the binding mode supposed for P5-S7A was also compared to that of atorvastatin in complex with HMG-CoAR (as reported in the PDB, accession code 1HWK [51]). The structural alignment of both complexes (Figure 9) permitted to us suppose that the first four residues of P5-S7A essentially reproduce the contact played by the three aromatic substituents of the atorvastatin pyrrole ring. In particular, the aniline is mimicked by the P5-S7A-Ile2 side chain, the P5-S7A-Leu3 was overlapped to the phenyl ring of the statin, and the *p*-F-phenyl ring of atorvastatin was spatially close to the P5-S7A-Pro4 (Figure 9). Unfortunately, the remaining moiety of the peptide pointed to an enzyme area different from the one in which the 3,5-dihydroxyl-heptanoic acid moiety was bound in the HMG-CoAR/atorvastatin complex. This portion is considered essential for the biological activity of the statins and could explain the reason on the base of the low affinity displayed by the mutant peptide. More efforts should be made to design peptides capable of mimicking such interactions and occupying the HMG-CoAR pocket sized by Lys735, Ser684, Arg590, Lys 691, Asn755, and Glu559 residues (Figure 9B).

## 4. Conclusions

In this study, using promising data on the dual hypocholesterolemic activity of the lupin peptide P5, we computationally designed new analogs endowed with improved PCSK9 and HMG-CoAR inhibitory activities. After the computational alanine-scanning mutational analysis, the non-hotspot residue of P5 was suitably substituted with other amino acids capable of improving the complementarity between PCSK9 and the peptide. Therefore, using our “affinity maturation protocol”, we selected P5-Best, P5-H6A, and P5-S7A peptides for the experimental assays. The attained experimental data confirmed the theoretical studies, revealing that the affinity of the mutant P5-H6A peptide on PCSK9 was reduced almost seven times (IC_50_ = 9.0 µM), whereas the affinity of P5-S7A was slightly higher than that of P5 (IC_50_ = 1.45 µM). Remarkably, the mutant peptide P5-Best showed the lowest PCSK9 IC_50_ value of 0.7 µM. Further biological assays demonstrated that all mutant peptides that maintained the dual PCSK9/HMG-CoAR inhibitory activity improved the ability of HepG2 cells to absorb extracellular LDL by up to 254% (P5-Best data). Doubtless, peptide P5 and its analogs display activity in the micromolar range suggesting that still their exploitation in the clinical application is challenging. Therefore, more efforts have to be pursued in order to improve their dual-inhibitory activity. However, evidences support the fact that P5 and its analogs can be considered as promising lead compounds for the development of a new class of hypocholesterolemic drugs endowed with dual-inhibitory activity of both PCSK9 and HMG-CoAR targets. Indeed, the dual and synergistic activity may be useful for better achieving the biological effect than compound actives on one of those targets.

Further experiments will be performed to evaluate the intestinal stability and propensity of P5 analogs to be trans-epithelial transported by mature Caco-2 cells. Experiments will be performed using the parent peptide P5 and the natural intestinal metabolite P5-met as positive controls. This study confirms that a multidisciplinary approach in the design of new peptides is successful in identifying peptides endowed with hypocholesterolemic effects, offering a promising starting point for the design of peptidomimetics that lack the bioavailability problems of peptides.

## Figures and Tables

**Table 1:** Estimated binding free energy values of the peptides under investigation, as calculated using the MM-GBSA approach (ΔG*, column 3).

Peptide/Mutation	Sequence	ΔG* Value ^1^	ΔΔG* Value ^2^
P5	LILPKHSDAD	−18.9 ± 0.5	0
L1A	AILPKHSDAD	−13.5 ± 0.5	+5.4
I2A	LALPKHSDAD	−20.9 ± 0.5	−1.0
L3A	LIAPKHSDAD	−6.6 ± 0.7	+12.3
P4A	LILAKHSDAD	−23.0 ± 0.7	−4.1
K5A	LILPAHSDAD	−14.1 ± 0.6	+4.8
H6A (P5-H6A)	LILPKASDAD	−1.2 ± 0.5	+17.7
S7A (P5-S7A)	LILPKHADAD	−19.3 ± 0.3	−0.4
D8A	LILPKHSAAD	−21.9 ± 0.3	−3.0
D10A	LILPKHSDAA	−19.6 ± 0.5	−0.7

^1^ (kcal/mol ± (Std. Err. of Mean)); ^2^ (kcal/mol).

**Table 2:** Estimated binding affinity values of newly designed P5 analogs (columns 1–2), calculated by Prime software (ΔAffinity, column 3) and standard MD/MM-GBSA calculations (ΔG* values, column 4).

Peptide	Sequence	ΔAffinity ^1^	ΔG* Value ^2^
P5	LILPKHSDAD		−18.9 ± 0.5
[I2P-A9R] P5	LPLPKHSDRD	−23.9	−26.3 ± 0.6
[I2M-A9R] P5	LMLPKHSDRD	−23.5	−26.3 ± 0.6
[I2R-A9R] P5	LRLPKHSDRD	−21.5	−30.3 ± 0.7
[I2Q-A9R] P5	LQLPKHSDRD	−21.0	−24.8 ± 0.8
[I2L-A9R] P5	LLLPKHSDRD	−20.7	−25.4 ± 0.6
[I2Y-A9R] P5 (P5-Best)	LYLPKHSDRD	−20.4	−41.7 ± 0.7
[I2H-A9R] P5	LHLPKHSDRD	−20.4	−24.6 ± 0.8
[I2T-A9R] P5	LTLPKHSDRD	−19.5	−28.0 ± 0.5
[I2F-A9R] P5	LFLPKHSDRD	−19.4	−19.2 ± 0.3
[I2E-A9R] P5	LELPKHSDRD	−19.1	−24.5 ± 0.6

^1^ (kcal/mol); ^2^ (kcal/mol ± (Std. Err. of Mean)).

## Data Availability

Not applicable.

## Supplementary Materials

The following supporting information can be downloaded at: https://www.mdpi.com/article/10.3390/pharmaceutics14030665/s1, Figure S1. RMSD plot of PCSK9/P5 complex, Figure S2. RMSD plot of PCSK9/P5-H6A complex, Figure S3. RMSD plot of PCSK9/P5-best complex, Figure S4. RMSD plot of HMG-CoAR/P5-S7A complex, Figure S5. RMSD plot of HMG-CoAR/P5 complex, Table S1. additional experimental details on the biological assays, peptide purity data (HPLC and MS traces).
