# Supplementary material for: Computational Design and Biological Evaluation of Analogs of Lupin Peptide P5 Endowed with Dual PCSK9/HMG-CoAR Inhibiting Activity
Source: Pharmaceutics. 2022 Mar 18;14(3):665. doi: 10.3390/pharmaceutics14030665 (PMC8951016; doi:10.3390/pharmaceutics14030665)
Supplement: Supplementary file 1 [file pharmaceutics-14-00665-s001.zip › pharmaceutics-1593031-supplementary.pdf]

pharmaceutics

Supplementary Materials: Computational Design and Biological Evaluation of Analogs of Lupin Peptide P5 Endowed with Dual PCSK9/HMG-CoAR Inhibiting Activity

Carmen Lammi, Enrico M. A. Fassi, Jianqiang Li, Martina Bartolomei, Giulia Benigno, Gabriella Roda, Anna Arnoldi, and Giovanni Grazioso

Table S1. Percentage of population of P5-S7A clusters identified by means of average-linkage method implemented in AmberTools20.

P5-S7A Peptide population	
**Cluster number**	**%**
1	73.1
2	11.7
3	5.7
4	3.4
5	2.2
6	1.6
7	1.1
8	0.9
9	0.1
10	0.1

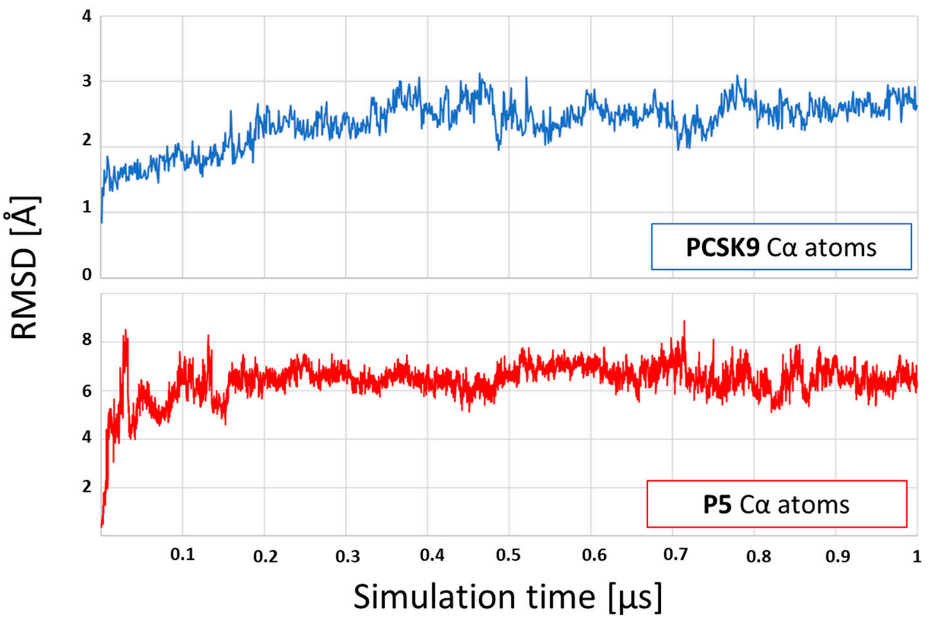

Figure S1. RMSD (Å) over simulation time of the complex PCSK9/**P5** Cα atoms aligned on the equilibrated structure of PCSK9.

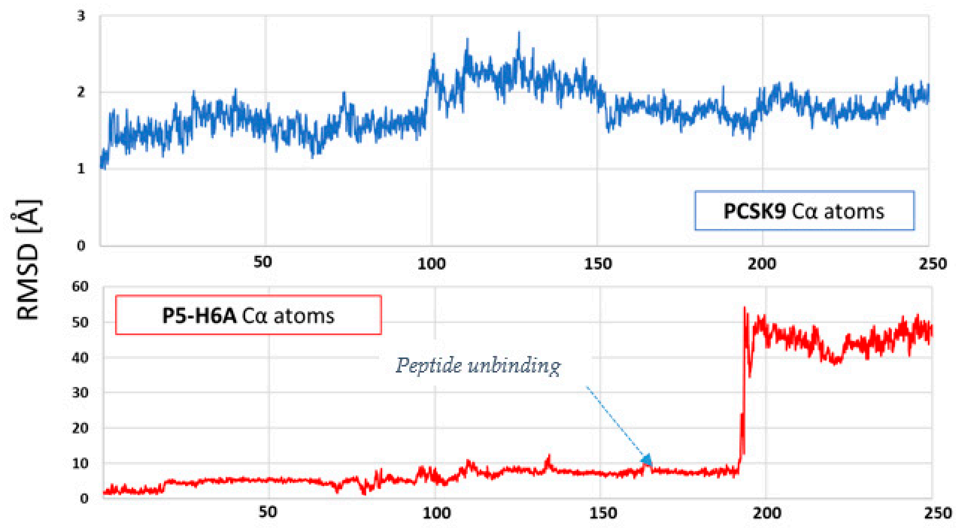

Figure S2. RMSD (Å) over simulation time of the complex PCSK9/**P5-H6A** Cα atoms aligned on the equilibrated structure of PCSK9.

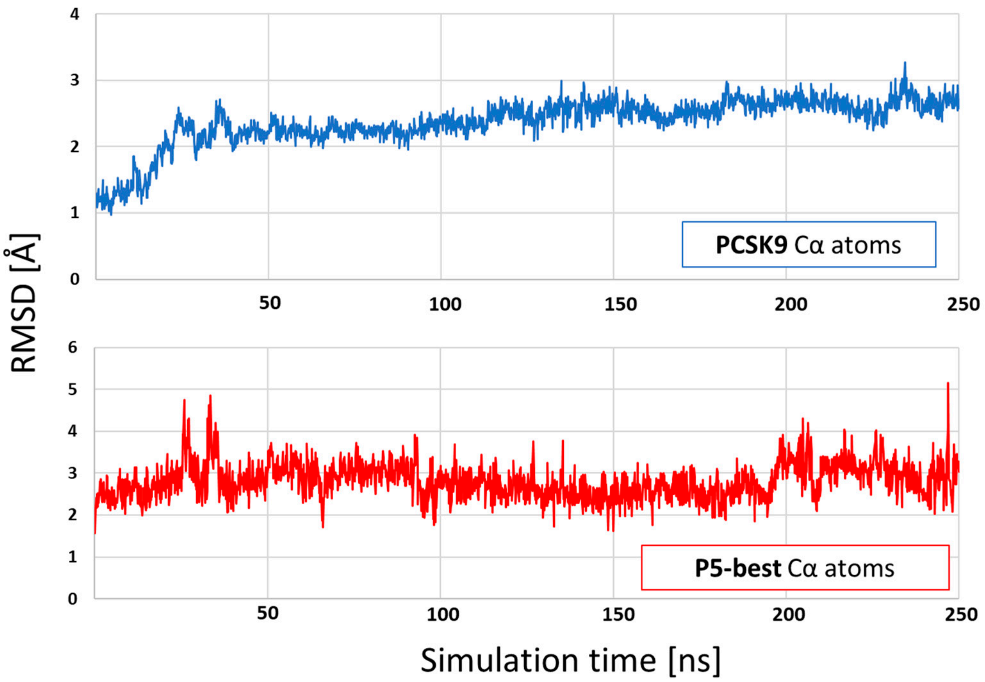

Figure S3. RMSD (Å) over simulation time of the complex PCSK9/**P5-best** Cα atoms aligned on the equilibrated structure of PCSK9.

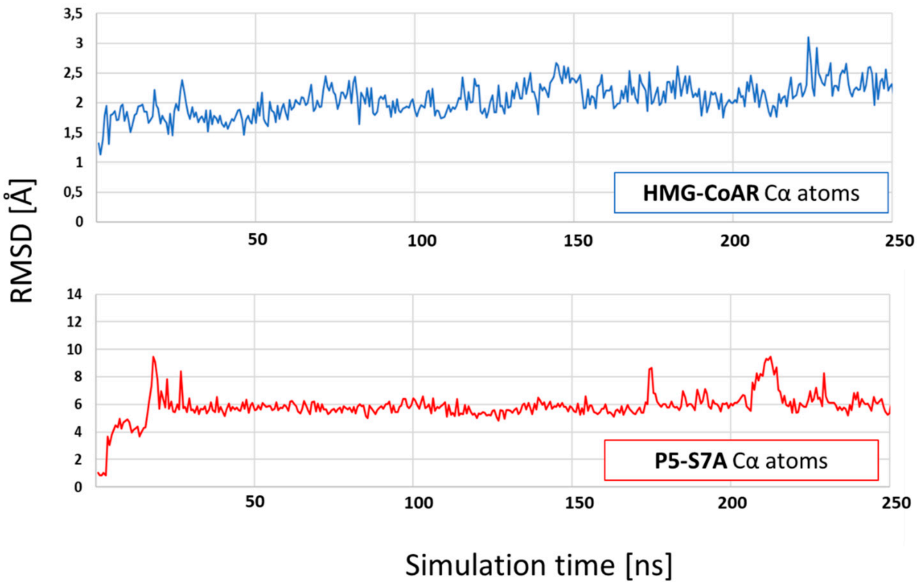

Figure S4. RMSD (Å) over simulation time of the complex HMG-CoAR/**P5-S7A** Cα atoms aligned on the equilibrated structure of HMG-CoAR.

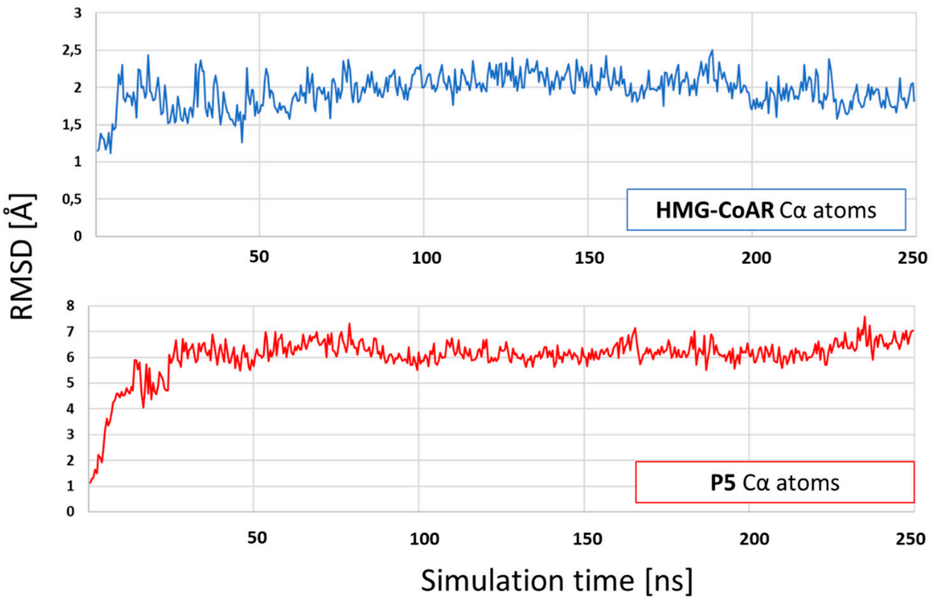

Figure S5. RMSD (Å) over simulation time of the complex HMG-CoAR/**P5** Cα atoms aligned on the equilibrated structure of HMG-CoAR.

Materials and Methods

Chemicals. Dulbecco's modified Eagle's medium (DMEM), stable L-glutamine, fetal bovine serum (FBS), phosphate buffered saline (PBS), penicillin/streptomycin, chemiluminescent reagent, and 96-well plates were purchased from Euroclone (Milan, Italy). The HMG-CoAR assay kit, bovine serum albumin (BSA), Janus Green B, formaldehyde, HCl and H_2SO_4 were from Sigma-Aldrich (St. Louis, MO, USA). The antibody against LDLR and the 3, 3', 5, 5'-tetramethylbenzidine (TMB) substrate were bought from Thermo Fisher Scientific (Waltham, MA, USA). The Quantikine ELISA kit was bought from R&D Systems (Minnneapolis, MN, USA). The LDL-DyLight™ 550 was from Cayman Chemical (Ann Arbor, MI, USA). The CircuLex PCSK9 *in vitro* binding Assay Kit was from CircuLex (Cy-

cLex Co., Nagano, Japan). The peptides (P5, P5-Best, P-H6A, and P5-S7A) were synthesized by the company GeneScript (Piscataway, NJ, USA) at >95% purity. The antibody against HMG-CoAR was bought from Abcam (Cambridge, UK). Phenylmethanesulfonyl fluoride (PMSF), Na-orthovanadate inhibitors, and the antibodies against rabbit Ig-horseradish peroxidase (HRP), mouse Ig-HRP, and SREBP-2 (which recognizes epitope located in a region between 833–1141 and bands at about 132 kDa) were purchased from Santa Cruz Biotechnology Inc. (Santa Cruz, CA, USA). The antibodies against hepatocyte nuclear factor 1-alpha (HNF1-alpha) and PCSK9 were bought from GeneTex (Irvine, CA, USA). The inhibitor cocktail Complete Midi was from Roche (Basel, Switzerland). Mini protean TGX pre-cast gel 7.5% and Mini nitrocellulose Transfer Packs were purchased from BioRad (Hercules, CA, USA).

HepG2 Cell Culture Conditions and Treatment. The HepG2 cell line was bought from ATCC (HB-8065, ATCC from LGC Standards, Milan, Italy) and was cultured in DMEM high glucose with stable L-glutamine, supplemented with 10% FBS, 100 U/mL penicillin, 100 μg/mL streptomycin (complete growth medium) with incubation at 37 °C under 5% CO_2 atmosphere.

HMG-CoAR Aactivity Assay. The experiments were carried out following the manufacturer instructions and optimized protocol [1]. The assay buffer, NADPH, substrate solution, and HMG-CoAR were provided in the HMG-CoAR Assay Kit (Sigma Aldrich SRL, Milan, Italy). The experiments were carried out following the manufacturer instructions at 37 °C. In particular, each reaction (200 μL) was prepared adding the reagents in the following order: 1 x assay buffer, a 10–500 μM doses of P5 and P5 analogs or vehicle (C), the NADPH (4 μL), the substrate solution (12 μL), and finally the HMG-CoAR (catalytic domain) (2 μL). Subsequently, the samples were mixed and the absorbance at 340 nm read by the microplate reader Synergy H1 (Winooski, VT, USA) at time 0 and 10 min. The HMG-CoAR-dependent oxidation of NADPH and the inhibition properties of peptides were measured by absorbance reduction, which is directly proportional to enzyme activity.

***In Vitro* PCSK9-LDLR Binding Assay.** Peptides P5 and P5 analogs (0.1–100 μM) were tested using the in vitro PCSK9-LDLR binding assay (CycLex Co., Nagano, Japan) following the manufacture instructions and conditions already optimized [2]. Briefly, plates are pre-coated with a recombinant LDLR-AB domain containing the binding site of PCSK9. Before starting the assay, tested peptides and/or the vehicle were diluted in the reaction buffer and added in microcentrifuge tubes. Afterwards, the reaction mixtures were added in each well of the microplate and the reaction was started by adding His-tagged PCSK9 solution (3 μL). The microplate was allowed to incubate for 2 h at room temperature (RT) shaking at 300 rpm on an orbital microplate shaker. Subsequently, wells were washed 4 times with the wash buffer. After the last wash, the biotinylated anti-His-tag monoclonal antibody (100 μL) was added and incubated at RT for 1 h shaking at 300 rpm. After incubation, wells were washed for 4 times with wash buffer. After the last wash, 100 μL of HRP-conjugated streptavidin were added and the plate was incubated for 20 min at RT. After incubation, wells were washed 4 times with wash buffer. Finally, the substrate reagent (tetra-methylbenzidine) was added, and the plate was incubated for 10 min at RT shaking at ca. 300 rpm. The reaction was stopped with 2.0 M sulfuric acid and the absorbance at 450nm was measured using the Synergy H1 fluorescent plate reader (Winooski, VT, USA).

In-Cell Western (ICW) Assay. For the experiments, a total of 3×10^4 HepG2 cells/well were seeded in 96-well plates. The following day, cells were washed with PBS and then starved overnight (O/N) in DMEM without FBS and antibiotics. After starvation, HepG2 cells were treated with 4.0 μg/mL PCSK9-WT and 4.0 μg/mL PCSK9 + peptides P5 and/or P5 analogs (50.0 μM) and vehicle (H_2O) for 2 h at 37 °C under 5% CO_2 atmosphere. Subsequently, they were fixed in 4% paraformaldehyde for 20 min at room temperature (RT). Cells were washed 5 times with 100 μL of PBS/well (each wash was for 5 min at RT) and the endogenous peroxides activity quenched adding 3% H_2O_2 for 20 min at RT. Non-specific sites were blocked with 100 μL/well of 5% bovine serum albumin (BSA, Sigma) in

PBS for 1.5 h at RT. LDLR primary antibody solution (1:3000 in 5% BSA in PBS, 25 μL/well) was incubated O/N at +4 °C. Subsequently, the primary antibody solution was discarded and each sample was washed 5 times with 100 μL/well of PBS (each wash was for 5 min at RT). Goat anti-rabbit Ig-HRP secondary antibody solution (Santa Cruz) (1:6000 in 5% BSA in PBS, 50 μL/well), was added and incubated 1 h at RT. The secondary antibody solution was washed 5 times with 100 μL/well of PBS (each wash for 5 min at RT). Freshly prepared TMB substrate (Pierce, 100 μL/well) was added and the plate was incubated at RT until desired color was developed. The reaction was stopped with 2 M H_2SO_4 and then the absorbance at 450 nm was measured using the microplate reader Synergy H1 (Winooski, VT, USA). After the read, cells were stained by adding 1 x Janus Green stain, incubating for 5 min at RT. The dye was removed, and the sample washed 5 times with water. Afterward 100 μL 0.5 M HCl for well were added and incubated for 10 min. After 10 seconds shaking, the OD at 595 nm was measured using the microplate reader Synergy H1 (Winooski, VT, USA).

Fluorescent LDL Uptake. HepG2 cells (3 x 10^4/well) were seeded in 96-well plates and kept in complete growth medium for 2 days before treatment. The third day, cells were washed with PBS and then starved overnight (O/N) in DMEM without FBS and antibiotics. After starvation, they were treated with 4.0 μg/mL PCSK9 and 4.0 μg/mL PCSK9 + P5 and/or P5 analogs (50.0 μM), and vehicle (H_2O) for 2 h with at 37 °C under 5% CO_2 atmosphere. At the end of the treatment, the culture medium was replaced with 50 μL/well LDL-DyLight™ 550 working solution (Cayman Chemical Company, Ann Arbor, MI, USA) prepared in DMEM without FBS and antibiotics. The cells were additionally incubated for 2 h at 37 °C and then the culture medium was aspirated and replaced with PBS (100 μL/well). The degree of LDL uptake was measured using the Synergy H1 fluorescent plate reader (Winooski, VT, USA) (excitation and emission wavelengths 540 and 570 nm, respectively). Fluorescent LDL-uptake was finally assessed following optimized protocol [3].

Western Blot Analysis. Immunoblotting experiments were performed using optimized protocol [3]. A total of 1.5 x 10^5 HepG2 cells/well (24-well plate) were treated with 50.0 μM of P5 and P5 analogs for 24 h. After each treatment, the supernatants were collected and stored at -20 °C; cells were scraped in 40 μL ice-cold lysis buffer (RIPA buffer + inhibitor cocktail + 1:100 PMSF + 1:100 Na-orthovanadate + 1:1000 β-mercaptoethanol) and transferred in ice-cold microcentrifuge tubes. After centrifugation at 13,300 *g* for 15 min at 4 °C, the supernatants were recovered and transferred into new ice-cold tubes. Total proteins were quantified by the Bradford's method and 50 μg of total proteins loaded on a pre-cast 7.5% Sodium Dodecyl Sulfate-Polyacrylamide (SDS-PAGE) gel at 130 V for 45 min. Subsequently, the gel was pre-equilibrated in H_2O for 5 min at room temperature (RT) and transferred to a nitrocellulose membrane (Mini nitrocellulose Transfer Packs,) using a Trans-Blot Turbo at 1.3 A, 25 V for 7 min. Target proteins, on milk or BSA blocked membrane, were detected by primary antibodies as follows: anti-SREBP-2, anti-LDLR, anti-HMG-CoAR, anti-PCSK9, anti HNF1-α and anti-β-actin. Secondary antibodies conjugated with HRP and a chemiluminescent reagent were used to visualize target proteins and their signal was quantified using the Image Lab Software (Biorad, Hercules, CA, USA). The internal control β-actin was used to normalize loading variations.

References

1. Aiello, G.; Lammi, C.; Boschin, G.; Zanoni, C.; Arnoldi, A. Exploration of Potentially Bioactive Peptides Generated from the Enzymatic Hydrolysis of Hempseed Proteins. *J. Agric. Food Chem.* **2017**, *65*, 10174–10184.
2. Lammi, C.; Zanoni, C.; Aiello, G.; Arnoldi, A.; Grazioso, G. Lupin peptides modulate the protein-protein interaction of PCSK9 with the low density lipoprotein receptor in HepG2 cells. *Sci. Rep.* **2016**, *6*, 29931.
3. Zanoni, C.; Aiello, G.; Arnoldi, A.; Lammi, C. Investigations on the hypocholesterolaemic activity of LILPKHSDAD and LTFPG-SAED, two peptides from lupin beta-conglutin: Focus on LDLR and PCSK9 pathways. *J. Funct. Foods* **2017**, *32*, 1–8.
